# Warm up and cool down!

**DOI:** 10.18632/oncotarget.5721

**Published:** 2015-09-18

**Authors:** Tao Wang, Dengke Ma

**Affiliations:** Cardiovascular Research Institute and Department of Physiology, UCSF School of Medicine, San Francisco, CA, USA

**Keywords:** Chromosome Section, temperature adaptation, membrane fluidity, Acyl-CoA dehydrogenase, fatty acid

In adaption to shifts in ambient temperature, both prokaryotic and eukaryotic cells maintain physiological homeostasis by adjusting various cellular properties, including membrane lipid fluidity, the capacity to maintain proteostasis and rate of protein synthesis [1]. How cells respond to temperature changes is a fundamental question in cell physiology. Here we briefly review current understanding of cellular adaptation to temperature shifts, focusing on our recent findings of homeostatic membrane fluidity regulation by the EGL-25/ACDH-11 pathway in *C. elegans* [2].

Several molecular sensors that transduce temperature shifts to cellular homeostatic responses have been previously described. Heat shock factor (HSF) is an evolutionarily conserved master regulator of heat shock-induced transcription. HSF is normally sequestered by cytoplasmic chaperon proteins and remains inactive. To alleviate protein mis-folding and subsequent aggregation upon heat stress, heat shock chaperon proteins bind to mis-folded proteins and dissociate from HSF. This allows HSF to translocate to the cell nucleus and activate transcription by binding to DNA regulatory elements to promote proteostasis and thermotolerance. Exposure to cold temperature also profoundly affects organismal physiology. For example, hypothermia protects cells against cellular oxidative injuries. Unlike the well-studied HSF pathway, how cold is sensed and transduced to downstream effectors remains poorly defined. In addition, molecules specifically present in thermosensory neurons can also sense temperature changes. The best-known molecular thermoreceptors are conserved members of the transient receptor potential (TRP) family of cation channels. For example, in mammals, TRPV1-4 and TRPM8 channels sense heat and cold, respectively. In *C. elegans*, TRPA-1 senses cold and mediates cold-induced extension of lifespan [3].

Because temperature shifts thermodynamically alter membrane fluidity in a nonspecific manner, cells must maintain membrane fluidity homeostasis upon shifts in temperature. Low temperature decreases the fluidity of the cytoplasmic membrane, which triggers upregulation of lipid desaturases to convert saturated fatty acids into unsaturated fatty acids to increase membrane fluidity [4]. This fundamental process of maintaining normal ranges of membrane fluidity is called homeoviscous adaptation (HVA). HVA occurs in single-celled as well as multicellular organisms. However, mechanisms for HVA in eukaryotes have been unknown.

From a *C. elegans* genetic screen exploring how genes control sensitivity to oxygen, Ma *et al.* discovered a novel pathway comprising the genes *egl-25* (or *paqr-2*, a homolog of mammalian adiponectin receptors) and *acdh-11* (acyl-CoA dehydrogenase, ACDH) that promote temperature adaptation via a stearoyl-CoA desaturase, FAT-7. *C. elegans* survives and reproduces optimally in temperatures ranging from 15°C to 25°C; this requires membrane fluidity to be maintained in normal ranges when temperature shifts. *egl-25* promotes adaptation of *C. elegans* to cold by regulating intestinal expression of *fat-7* [2, 5]. Ma *et al.* identified 8 loss-of-function mutations of a previously uncharacterized gene *acdh-11* that rescued the *egl-25* mutant phenotype. *acdh-11* encodes an acyl-CoA dehydrogenase, and acts downstream of *egl-25* to mediate signaling through medium-chain C11/C12 fatty acids. By biochemical studies and crystal structure analysis of ACDH-11, Ma *et al.* further showed that ACDH-11 inhibits *fat-7* expression by sequestering C11/C12− chain fatty acids, therefore inactivating nuclear hormone receptors, likely NHR-49 (a *C. elegans* homolog of the mammalian fatty acid-binding transcription factors HNF4a and PPARα) and preventing them from activating *fat-7* expression. In response to cold (15°C), cells upregulate *fat-7* to promote lipid desaturation and thus increase membrane fluidity. This study suggests that intracellular C11/C12 fatty acids promote *fat-7* expression via binding to nuclear receptors including NHR-49. In response to heat (25°C), elevated levels of the ACDH-11 protein sequester intracellular C11/C12 to inhibit *fat-7* expression, thereby promoting lipid saturation and decrease membrane fluidity.

This study raises several important unanswered questions concerning the detailed mechanism of HVA. For example, upstream sensors and mediators of heat-induced *acdh-11* remain unknown. Since heat adaption can be mediated by heat-shock factors HSF-1 and HSF-2, it is possible that the upregulation of *acdh-11* depends on HSF-1/2. Furthermore, *C. elegans egl-25* mutants fail to adapt to cold, mechanisms of which remain to be elucidated. *egl-25* encodes a *C. elegans* homolog of the evolutionarily conserved family of PAQR receptors. Since adiponectin is markedly induced by cold exposure in humans, one possibility is that EGL-25 constitutes part of the cold-sensing pathway as a receptor for cold-inducible ligand expression. Additional genetic analysis of the EGL-25/ACDH-11 pathway will likely help identify novel mechanisms of temperature-sensing and homeostatic responses to temperature shifts.

Roles of EGL-25, ACDH-11 and FAT-7 might be evolutionarily conserved in many other organisms. ADIPOR1 and ADIPOR2, two homologs of *C. elegans* EGL-25, are abundant in adipocytes, endothelial cells and cardiomyocytes. In humans, cold exposure increases levels of circulating adiponectin independently of changes in core body temperatures, and ADIPORs are known to be involved in regulating fatty acid signaling, suggesting a potential link between temperature and fatty acid regulation in mammals [6]. Given lipid metabolism and signaling are fundamental cellular functions that are similar between nematodes and other organisms, and EGL-25, ACDH-11, FAT-7 have homologs in mammals (AdipoR1/2-VLCAD-FADS1,2,3 in humans), it is likely that regulatory principles of the EGL-25-ACDH-11- FAT-7 axis for temperature adaptation are evolutionarily conserved.
